# Fulminant Neonatal Liver Failure in MPV 17-Related Mitochondrial DNA Depletion Syndrome

**DOI:** 10.1155/2023/4514552

**Published:** 2023-06-20

**Authors:** Razan Abduljalil, Hadhami Ben Turkia, Aysha Fakhroo, Cristina Skrypnyk

**Affiliations:** ^1^Department of Pediatrics, King Hamad University Hospital, Manama, Bahrain; ^2^Department of Molecular Medicine, Al‐Jawhara Centre for Molecular Medicine, Arabian Gulf University, Manama, Bahrain

## Abstract

Mitochondrial depletion syndromes are well established causes of liver failure in infants. Hepatocerebral variant related to MPV17 gene defect is characterized by infantile onset of progressive liver failure, developmental delay, neurological manifestations, lactic acidosis, hypoglycemia, and mtDNA depletion in liver tissue. We report a hepatocerebral variant of mitochondrial DNA depletion syndrome in a neonate who presented with septic shock picture, hypoglycemia, jaundice, hypotonia, and rotatory nystagmus. Family history was significant for consanguinity and a brother who died at the age of 4 months. Investigations showed mild liver function derangement contrasting with severe coagulopathy, hyperlactatemia, and generalized aminoaciduria. The brain MRI was normal. Next generation sequencing (NGS) panel identified a MPV17 gene missense homozygous pathogenic variant. The infant expired at the age of 2 weeks with refractory ascites. This case illustrates a challenging diagnosis causing liver failure and death in neonatal period. Genetic testing of mitochondrial DNA depletion syndromes should be a part of liver failure workup in addition to other treatable disorders presenting with encephalo-hepatopathy in infancy.

## 1. Introduction

Neonatal liver failure (NLF) is a rare and life-threatening condition. Viral infections, gestational alloimmune liver disease (GALD), and metabolic diseases are the main causes leading to NLF [1, 2]. Galactosemia, mitochondrial disorders in particular mitochondrial DNA maintenance defects (MDMDs), and tyrosinemia type 1 are the most prevalent causes of liver failure in infancy [2, 3].

MDMDs are rare disorders that result from nuclear DNA genes mutations affecting the maintenance of mitochondrial DNA (mtDNA) leading to severe reduction of mtDNA content and insufficient synthesis of respiratory chain complexes in different tissues. MDMDs are genotypically and phenotypically heterogeneous group of autosomal recessive disorders that affect either a specific organ or a combination of organs, including muscle, liver, brain, and kidney, delineating different phenotypes such as myopathic, encephalomyopathic, neurogastrointestinal, and hepatocerebral [4–6]. The hepatocerebral phenotype is caused by homozygous or compound heterozygous mutations in DGUOK (OMIM 251880), MPV17 (OMIM 256810), POLG1 (OMIM 174763), TFAM (OMIM 617156), or TWNK (OMIM 271245) nuclear genes [6, 7]. The clinical presentation varies from subtle hepatic dysfunction to early onset of liver failure with or without neurologic manifestations making the diagnosis challenging [8, 9].

MPV17 gene is coding for the MPV17 protein, a mitochondrial inner membrane protein involved in importing deoxynucleotides into the mitochondria [6]. The majority of affected individuals reported with MPV17 gene variant had an early onset hepatic and neurological manifestations, failure to thrive, lactic acidemia, and mtDNA depletion detected mainly in liver tissue [6].

Herein, we report a fulminant NLF related to MPV17 gene homozygous pathogenic variant in a newborn. Clinical and laboratory data were collected from the medical records. Extended literature review was done on the previous reported cases with MPV17 gene variant. The research protocol was approved by the Research Ethical Committee of King Hamad University Hospital. Individual written consents for publication were obtained from the parents.

## 2. Case Presentation

A 6-day-old baby girl presented with lethargy and poor oral intake. She was born to Syrian consanguineous parents at 41 weeks, with a birth weight of 2.75 kg. The family history was significant for a brother who died at the age of 4 months because of LF. The other five siblings were healthy. She was exclusively breastfed. She was found to have poor perfusion, jaundice, and hypoglycemia (1 mmol/l). Neither hepatosplenomegaly nor dysmorphic feature was identified, and the cardiovascular examination was normal. She was severely hypotonic with weak cry, and intermittent roving eye movements were observed on pull to sit maneuver. The brain MRI was normal. A tuberous hemangioma was noted on the right hand.

The baby was started on respiratory and hemodynamic support, antibiotics, intravenous dextrose infusion, carnitine, and multivitamin cocktail.

Detailed blood (Table 1) and imaging investigations were performed and showed.

Hyperlacticacidemia, mildly deranged liver function test, sever coagulopathy not improving after vitamin K, negative septic workup, and negative TORCH infection. Hyperlacticacidemia, mildly deranged liver function test, sever coagulopathy not improving after vitamin K, negative septic workup, and negative TORCH infection. Metabolic workup showed nonspecific increase of plasma arginine, methionine, and phenylalanine due to liver failure. Urinary organic acids were positive for lactic acid, dicarboxylic acids, and tyrosine metabolites. Urine aminoacids chromatography showed generalized aminoaciduria. GALT activity and plasma acylcarnitines profile were normal. The liver and the spleen were normal in size, shape, and echo pattern; a hyperechoic solid focal lesion was seen in right lobe (suspecting hemangioma). Echocardiogram was normal and brain MRI did not show any anomalies. The baby remained hypotonic and developed refractory ascites. The lactatemia was fluctuating reaching 16 mmol/l. Despite full support, the baby expired at the age of 2 weeks. Next generation sequencing (NGS) panel (targeted regions coverage 99.6%, sequencing depth >30x, Beijing Genome Institute Clinical laboratory) covering 15 genes associated with MDMDs.

Next generation sequencing (NGS) panel covering 15 genes associated with MDMDs (DGUOK, C10ORF2, MPV17, POLG, POLG2, RRM2B, SUCLA2, SUCLG1, TK2, OPA1, OPA3, TYMP, AARS2, APTX, and SLC25A4) revealed a previously reported homozygous missense variant c.278A>C (p.Gln93Pro) in exon 4 of MPV17 gene. The variant leads to a substitution in a highly conserved amino acid domain and is assessed as pathogenic, disease-causing mutation (class1).

## 3. Discussion

Mitochondrial disorders are well established causes of acute LF in infants, reported in around 20% in the different studies [8–10]. MPV17 gene encodes a mitochondrial inner membrane controlling mtDNA maintenance and OXPHOS activity. The mechanisms through which MPV17 mutations cause respiratory chain dysfunction and mtDNA depletion are still unclear [11]. Different mutations lead to different cellular abnormalities, including increased reactive oxygen species production, decreased oxygen consumption, loss of mitochondrial membrane potential, and mislocalization of MPV17 protein [11].

Pathogenic variants in MPV17 were first reported in 2006 to cause a hepatocerebral MDS characterized by infantile onset of progressive liver failure, developmental delay, neurological manifestations, lactic acidosis, hypoglycemia, and mtDNA depletion in liver tissue [12]. In 2014, Alhussaini reported that 22% of infantile LF cases had MPV17 and DGUOK pathogenic variants [13].

A review of 100 individuals with MPV17-related MDMDs reported that 96% of patients presented with the infantile hepatocerebral phenotype [6]. Our patient presented with a fulminant LF associated with severe hypotonia since the 1^st^ week of life. In El-Hattab cohort, clinical manifestations started during neonatal period in 38% and within the 1^st^ year of life in 58% of the patients. Ninety-one % of these patients progressed to LF in infancy or early childhood. Some patients displayed psychomotor delay during early infancy while others have normal development early in life followed by loss of motor and cognitive abilities later in infancy or early childhood [6]. Twelve patients were reported with the same MPV17 gene pathogenic variant (p.Gln93Pro) [6, 14, 15]. There was no real difference between the phenotype and the outcome of all the patients. All of them died after the age of 5 months; our patient did not survive beyond the age of 2 weeks. Hepatocerebral MDMDs diagnosis can be missed in the neonatal period since liver dysfunction and neurological impairment are common in severely sick newborn [5]. Our patient presented with shock, acute encephalopathy, hypoglycemia, and high lactate level that are all suggestive of sepsis. Mild increase of liver enzymes contrasting with the severity of the coagulopathy, hypoglycemia, and positive family history of neonatal death pointed to a metabolic disorder. The presence of hypotonia associated with the rotatory oscillating eye movements and the persistent hyperlactacidemia pointed towards MDMDs. Even though nystagmus was reported in only 7% of MPV17-MDMDs affected individuals [6], this sign can guide the clinical diagnosis towards a mitochondrial disease. MPV17 phenotype is indistinguishable from many other treatable disorders such as tyrosinemia type 1 which was unlikely in our patient given the age at presentation, galactosemia, GALD, infectious conditions, and hemophagocytic lymphohistiocytosis that should be ruled out [2, 7, 8, 16]. The availability of the NGS testing targeting particular mitochondrial depletion syndrome spectrum allowed a fast diagnosis and helped further for the accurate genetic counseling of the parents.

Furthermore, high level of tyrosine, alpha fetoprotein, and ferritin were reported in DGUOK MDMDs resembling tyrosinemia 1 or GALD. Renal tubular dysfunction is also found in galactosemia, tyrosinemia, and fructose intolerance. High plasma lactate and significant clue, although not specific, were reported in 79% of El-Hattab cohort [6]. Normal lactate does not exclude the diagnosis.

As seen in our patient, infants with MPV17-MDMDs could have normal brain MRI despite evident neurologic impairment. The most common observed MRI abnormality is diffused white matter abnormalities noted in 38% of patients reported by El-Hattab. Abnormal lesions might appear after infancy. MRI follow up is indicated if liver transplantation is considered, but our patient did not survive to approach this therapy. However, even for those patients who survived the neonatal period, the management remains supportive, and the outcome of liver transplantation is poor because of the multisystem involvement in this disorder [6].

MPV17-MDMDs are likely to be rare but probably underestimated in areas with high rate of consanguinity. Majority of the MPV17 pathogenic variants are private. The homozygous c.278A>C (p.Gln93Pro) missense mutation has been identified in 12 individuals mainly from Arab families suggesting a possible founder effect. This is, to our best knowledge, the first reported patient of Syrian origin [16].

## 4. Conclusion

The prevalence of MDMDs disorders is probably underestimated due to their clinical heterogeneity. The phenotype of MPV17-related mtDNA maintenance defect is indistinguishable from many other inherited disorders presenting with encephalo-hepatopathy; hence, physician must have a high index of suspicion for these diseases to initiate as fast as possible the genetic testing.

## Figures and Tables

**Table 1:** Investigations.

Blood gas	pH 7.346, PCO2 37.8, HCO3^−^ 20, BE -5.5, anion gap 19.7
Lactate	9.4 mmol/l fluctuating up to 16 mmol/l
Glucose	1 mmol/l
Blood ketones	0.3 mmol/L
Liver function test	Albumin: 24 g/l, total/direct bilirubin: 213/42 *μ*mol/l, alanine aminotransferase (ALT)/aspartate aminotransferase (AST): 52/92IU/L G-glutamyltransferase (GGT): 496 *μ*mol/l, alkaline phosphatase: 291 *μ*mo/l
Ammonia	127 micromol/l and repeated normal
Coagulation study	PT 38 seconds, APTT 102 seconds, and INR: 3.4
CBC	WBCs 9.7, Hb 19.9, Ht 60, MCV 113, MCH 37.4, and platelets 245
Sepsis workup (blood, urine)	Negative
Tandem mass spectrometry on dried blood spots	Increases levels of arginine, ornithine, methionine, phenylalanine, and tyrosine
Plasma acyl-carnitines profile	Normal
Urinary GCMS organic acids	Mild to significant elevation of: (i) Lactic acid, 2-hydroxy butyric acid, 3-hydroxy butyric acid, and 2-hydroxy isovaleric acid (ii) Adipic acid, 2-hydroxy sebacic acid, 3-hydroxy sebacic acid, and 3-hydroxy dodecanedioic acid (dicarboxylic acids and their metabolites) (iii) 4-hydroxy phenylacetic acid, 4-hydroxy phenyllactic acid, and 3-hydroxy phenylpyruvic acid (tyrosine metabolites) (iv) Phenyllactic acid, phenylpyruvic acid-2-hydroxy adipic acid, and 2-ketoadipic acid
Urinary GCMS aminoacids	High levels of alanine, ethanolamine, glycine, phenylalanine, proline, serine, threonine, and tyrosine
Galactose-1-phosphate uridylyltransferase (GALT) activity	Normal
Serum ferritin	315 ng/ml (normal)

## Data Availability

The dataused to support the findings of this study are available at https://www.ncbi.nlm.nih.gov/clinvar/variation/694362/?oq=c.278A%3EC%5bvarname%5d+MPV17&m=NM_002437.5(MPV17): c.278A%3EC%20(p.Gln93Pro).
